# Multiple SNPs in Intron 41 of Thyroglobulin Gene Are Associated with Autoimmune Thyroid Disease in the Japanese Population

**DOI:** 10.1371/journal.pone.0037501

**Published:** 2012-05-25

**Authors:** Yoshiyuki Ban, Teruaki Tozaki, Matsuo Taniyama, Luce Skrabanek, Yasuko Nakano, Yoshio Ban, Tsutomu Hirano

**Affiliations:** 1 Division of Diabetes, Metabolism, and Endocrinology, Department of Internal Medicine, Showa University School of Medicine, Shinagawa, Tokyo, Japan; 2 Department of Pharmacogenomics, Showa University School of Pharmacy, Shinagawa, Tokyo, Japan; 3 Division of Endocrinology and Metabolism, Showa University Fujigaoka Hospital, Yokohama, Kanagawa, Japan; 4 Department of Physiology and Biophysics and HRH Prince Alwaleed Bin Talal Bin Abdulaziz Alsaud Institute for Computational Biomedicine, Weill Medical College of Cornell University, New York, New York, United States of America; 5 Ban Thyroid Clinic, Meguro, Tokyo, Japan; Instituto de Higiene e Medicina Tropical, Portugal

## Abstract

**Background:**

The etiology of the autoimmune thyroid diseases (AITDs), Graves' disease (GD) and Hashimoto's thyroiditis (HT), is largely unknown. However, genetic susceptibility is believed to play a major role. Two whole genome scans from Japan and from the US identified a locus on chromosome 8q24 that showed evidence for linkage with AITD and HT. Recent studies have demonstrated an association between thyroglobulin (Tg) polymorphisms and AITD in Caucasians, suggesting that Tg is a susceptibility gene on 8q24.

**Objectives:**

The objective of the study was to refine Tg association with AITD, by analyzing a panel of 25 SNPs across an extended 260 kb region of the Tg.

**Methods:**

We studied 458 Japanese AITD patients (287 GD and 171 HT patients) and 221 matched Japanese control subjects in association studies. Case-control association studies were performed using 25 Tg single nucleotide polymorphisms (SNPs) chosen from a database of the Single Nucleotide Polymorphism Database (dbSNP). Haplotype analysis was undertaken using the computer program SNPAlyze version 7.0.

**Principal Findings and Conclusions:**

In total, 5 SNPs revealed association with GD (P<0.05), with the strongest SNP associations at rs2256366 (P = 0.002) and rs2687836 (P = 0.0077), both located in intron 41 of the Tg gene. Because of the strong LD between these two strongest associated variants, we performed the haplotype analysis, and identified a major protective haplotype for GD (P = 0.001).These results suggested that the Tg gene is involved in susceptibility for GD and AITD in the Japanese.

## Introduction

Autoimmune thyroid diseases (AITDs), including Graves' disease (GD) and Hashimoto's thyroiditis (HT), are among the most common human autoimmune diseases. The prevalence in Caucasians is 1% [1], [2], and the prevalence in Japanese may be similar. GD is characterized clinically by hyperthyroidism, diffuse goiter and the presence of thyrotropin receptor (TSHR) antibodies. Some patients develop extrathyroidal manifestations, mainly ophthalmopathy and dermopathy (reviewed by Davies [3]). HT is characterized by apoptosis of thyrocytes leading to hypothyroidism (reviewed by Weetman [4]). However, despite their contrasting clinical presentations, GD and HT share many common features, mainly the infiltration of the thyroid by T cells and the production of anti-thyroid autoantibodies (anti-thyroglobulin and anti-thyroid peroxidase antibodies) [3]–[5].

The pathogenesis of AITDs is thought to involve several risk factors, including genetic risk factors (reviewed in [6]) and environmental triggers such as cigarette smoking, iodine intake and infection [7], [8] (reviewed by Davies [3]). However, the evidence for interactions between hereditary factors and environmental influences appears to be much stronger for cigarette smoking and iodine intake than for infections [8].

The first locus shown to be associated with AITDs was the HLA-DRB1 locus (reviewed in [6]). HLA-DR3 (DRB1*03) has been consistently shown to be associated with GD in Caucasians, with an odds ratio (OR) of 2.0–3.0 [9]–[11]. Other HLA alleles have been shown to be associated with GD in non-Caucasian populations (for a review see [12]). Non-HLA genes have also been shown to influence the expression of GD. These genes include the genes for CTLA-4 [13], CD40 [14], CD25 [15], thyroglobulin (Tg) [16] and TSHR [17], [18].

Two whole genome scans one from Japan [19] and one from the US [20] identified a locus on chromosome 8q24, which showed evidence for linkage with AITD and HT. The 8q24 locus contains the thyroglobulin (Tg) gene, one of the major autoantigens in AITD, and, thus, the Tg gene is a strong positional candidate gene for AITD in this locus [21]. Indeed, the microsatellite Tgms2, located inside intron 27 of the Tg gene, showed evidence for linkage (LOD score = 2.9) and association (p = 0.004) with AITD in a US dataset [16]. These results have been replicated in a UK dataset [22], showing a significant association between Tgms2 and AITD (p<0.001). Moreover, the same Tgms2 allele was found to be associated with AITD in both studies. Following these findings the entire Tg gene was sequenced and , case control association studies for 14 novel Tg single nucleotide polymorphisms (SNPs) in AITD patients and controls showed that one SNP cluster (the exons 10–12 cluster) and an exon 33 SNP were significantly associated with AITD [23]. Recently, we showed a significant association between Tgms2 and HT in an independent Japanese cohort [24]. In the present study, we performed a case-control study of AITD using 25 SNPs from the Single Nucleotide Polymorphism Database (dbSNP) databases spaced approximately 10–50 kb apart and spanning the Tg gene. We found significant associations between SNPs in intron 41 and GD.

## Materials and Methods

### Ethics Statement

The research protocol was approved by the Ethic Committee of the Showa University Hospital and each subject signed the informed consent form approved by the Institutional Review Board at the Showa University Hospital.

### Patients and Controls

#### AITD Patients

Four hundred and fifty-eight unrelated Japanese AITD patients were studied. There were a total of 287 GD patients and 171 HT patients.

#### Clinical assessment

GD was diagnosed base on clinical symptoms and biochemical confirmation of hyperthyroidism, including diffuse goiter, elevated radioactive iodine uptake, and elevated thyroid hormone levels. HT patients had documented clinical and biochemical hypothyroidism requiring thyroid hormone replacement therapy and showed autoantibodies against thyroid peroxidase with or without antibodies against thyroglobulin.

#### Controls

Two hundred and twenty-one age- and sex-matched healthy Japanese volunteers served as controls in our association studies. All controls had no personal or family history of any autoimmune disease.

### SNP typing

SNP selection was based on the HapMap Linkage disequilibrium (LD) blocks of the TG gene in Japanese so that the entire gene had covereage [25]. Twenty-five intronic SNPs, many of which were in the relationships without strong LD, in the Tg gene were chosen from a database of dbSNP (**Table S1**). DNA was extracted from whole blood using the Puregene kit (Gentra Systems, Minneapolis, MN). All SNPs were genotyped by the high-resolution melting and unlabeled probe methods using LightScanner® (Idaho Technology Inc., Salt Lake City, Utah) based on the manufacture's protocol.

### Statistical analysis

Case-control analysis and Hardy-Weinberg equilibrium (HWE) test of SNP were performed using SNPALyze ver. 7.0 (Dynacom, Yokohama, Japan) [26]. Differences in the allele frequencies between the groups were analyzed using the chi square test and Fisher's exact test. The odds ratio (OR) was calculated using the modified method of Woolf [27]. A p-value of <0.05 was considered statistically significant. HWE tests were carried out for all loci among subjects and controls separately. Tests in subjects and controls did not show any significant deviation from HWE for any of the SNPs. Linkage disequilibrium (LD) between SNPs was evaluated by the r^2^ of pair-wise LD using SNPALyze ver. 7.0 (Dynacom, see **Table S1**). Haplotype frequencies for multiple loci were estimated by phase estimation using the expectation-maximization (EM) algorithm. Permutation p values were calculated by comparing haplotype frequencies between cases and controls on the basis of 10,000 replications using SNPALyze ver. 7.0.

## Results

### Case-control study

All cases and controls were in Hardy Weinberg Equilibrium. **Table 1** shows frequencies of these alleles in patients and controls and the results of the case-control association analysis of alleles of 25 SNPs. With rs3739266 and rs2687836, we found significant differences between allele frequencies in subjects and controls, which were reflected in an increased frequency of the minor alleles in AITD and GD (P<0.05), compared with frequencies in controls. The evidence for association appears to be driven by associations with GD. When allele frequencies were compared between GD subjects and controls, all 4 SNPs in the interval rs3739266 and rs2687836 showed evidence of association. In contrast, there was little or no evidence of association between these SNPs and HT alone. The strongest SNP associations are all located within intron 41 of the Tg and are separated by just 5 kb; these include rs2256366 (P = 0.002) and rs2687836 (P = 0.0077) (**Table 2**).

**Table 1:** Allele frequencies and association analyses between SNPs in Japanese AITD patients and controls.

SNP No.	SNP Name	Controls (n = 221)	AITD (n = 458)		GD (n = 287)		HT (n = 171)	
		Minor allele frequency	Minor allele frequency	P value^a^ (OR)	Minor allele frequency	P value^a^ (OR)	Minor allele frequency	P value^a^ (OR)
[01]	−1984	0.25	0.25	0.96	0.25	0.89	0.25	0.93
[02]	−1714	0.47	0.49	0.42	0.48	0.63	0.51	0.28
[03]	rs180223	0.26	0.24	0.37	0.24	0.58	0.22	0.25
[04]	rs2069550	0.27	0.23	0.092	0.24	0.3	0.2	**0.032** (0.68)
[05]	rs853326	0.27	0.23	0.14	0.25	0.39	0.21	0.05
[06]	rs2068128	0.35	0.32	0.35	0.32	0.26	0.34	0.74
[07]	rs2261147	0.38	0.35	0.28	0.33	0.16	0.37	0.75
[08]	rs2069556	0.38	0.34	0.18	0.34	0.17	0.35	0.38
[09]	rs853304	0.38	0.33	0.13	0.33	0.15	0.34	0.26
[10]	rs2246624	0.19	0.19	0.83	0.21	0.53	0.18	0.62
[11]	rs2069561	0.38	0.35	0.41	0.36	0.49	0.35	0.43
[12]	rs7829428	0.44	0.47	0.35	0.46	0.5	0.48	0.29
[13]	rs2687809	0.21	0.22	0.67	0.22	0.71	0.22	0.71
[14]	rs2076740	0.22	0.25	0.24	0.26	0.19	0.24	0.52
[15]	rs10505604	0.22	0.26	0.15	0.26	0.19	0.26	0.24
[16]	rs3739266	0.2	0.26	0.021 (1.4)	0.26	0.043 (1.4)	0.27	0.04
[17]	rs2256366	0.44	0.51	0.019 (1.3)	0.54	0.002 (1.5)	0.46	0.63
[18]	rs2253035	0.18	0.22	0.073	0.23	0.037 (1.4)	0.2	0.43
[19]	rs2687836	0.4	0.45	0.057	0.48	0.0077 (1.4)	0.41	0.87
[20]	rs2252696	0.19	0.18	0.66	0.2	0.73	0.15	0.13
[21]	rs1124527	0.37	0.39	0.43	0.42	0.12	0.35	0.55
[22]	rs2256476	0.35	0.37	0.43	0.39	0.17	0.34	0.72
[23]	rs2069568	0.33	0.33	0.94	0.36	0.39	0.29	0.25
[24]	rs2069569	0.21	0.21	0.95	0.23	0.48	0.18	0.34
[25]	rs2294024	0.11	0.11	0.72	0.12	0.49	0.1	0.8

Bold indicates major allele frequencies that are significantly increased in cases (P<0.05). Underline indicates minor allele frequencies that are significantly increased in controls (P<0.05).

a P-value based on χ^2^ distribution.

**Table 2:** Case–control association results for the two most highly GD associated SNPs.

SNP Name	Allele/Genotype	Control (n = 221)	AITD (n = 458)	P value^a^	GD (n = 287)	P value^a^	HT (n = 171)	P value^a^
rs2256366	T	248 (56.1)	452 (49.3)		266 (46.3)		452 (54.4)	
	C	194 (43.9)	464 (50.7)	0.019	308 (53.7)	0.002	464 (45.6)	0.63
	T/T	72 (32.6)	108 (23.6)		59 (20.6)		108 (28.7)	
	T/C	104 (47.1)	236 (51.5)		148 (51.6)		236 (51.5)	
	C/C	45 (20.4)	114 (24.9)	0.04	80 (27.9)	0.0056	114 (19.9)	0.65
rs2687836	C	265 (60.0)	499 (54.5)		296 (51.6)		203 (59.4)	
	T	177 (40.0)	417 (45.5)	0.057	278 (48.4)	0.0077	139 (40.6)	0.87
	C/C	81 (36.7)	131 (28.6)		73 (25.4)		58 (33.9)	
	C/T	103 (46.6)	237 (51.7)		150 (52.2)		87 (50.9)	
	T/T	37 (16.7)	90 (19.7)	0.1	64 (22.3)	0.019	26 (15.2)	0.7

Values given are the number of subjects, with the percentage in parentheses.

a P-value based on χ^2^ distribution.

### Tg haplotype analysis

Because of the strong LD between the strongest two variants (*r^2^* = 0.793, see Table 1), haplotype analysis was undertaken using the computer program SNPAlyze version 7.0 (**Table 3**). Two haplotyes (haplotypes #1 and #2) were relatively common and haplotype #3 was rare. Distribution of the haplotype is significantly different between GD and control by permutation procedure (p = 0.018). Haplotype #2, which contained the both two SNPs' risk alleles, was found to be positively associated with GD and AITD (P = 0.003 for GD, P = 0.034 for AITD). In contrast, haplotype #1, which did not contain the both two SNPs' risk allele, was found to be protective (P = 0.001 for GD, P = 0.025 for AITD) (**Table 3**).

**Table 3:** Tg haplotype structure and frequencies^a^.

	SNP ID		Haplotype comparison^b^						
Haplotype	18	20	AITD	GD	HT	Controls	AITD vs Controls	GD vs Controls	HT vs Controls
							P-value	P-value	P-value
1	T	C	0.49	0.46	0.54	0.55	0.025	0.001	0.73
2	C	T	0.45	0.48	0.4	0.39	0.034	0.003	0.8
3	T	T	0.055	0.056	0.053	0.046	0.47	0.45	0.69

a The program, SNPAlyze ver. 7.0 Standard, was used to estimate common (frequencies >0.01) haplotypes for the two SNPs genotyped.

b Each haplotype was compared with the other haplotypes combined.

## Discussion

We performed a case-control study using 25 SNPs located in the Tg gene to test association with AITD, GD, and HT. We found a significant association between AITD/GD/HT with several SNPs, with strongest SNP associations at rs2256366 (P = 0.002) and rs2687836 (P = 0.0077), both located in intron 41 of the Tg gene (Tables 1 and 2). The Tg gene region has been previously shown to be linked with AITD in a Japanese dataset of sib-pairs [19]. However, polymorphisms in the Tg gene itself have not been previously studied for association with AITD in the Japanese. Recently, amino acid sequence variants in the Tg gene were reported to be associated with AITD [23]. In a Caucasian US cohort one SNP cluster (the exons 10–12 cluster) and an exon 33 SNP were significantly associated with AITD [23]. Recently, One from Taiwanese [28] found a significant increase in the T/T genotype of the exon 33 SNP compared with the control group (P<0.001). However, our preliminary data in Japanese HT patients and controls showed that the exon 33 SNP of the Tg gene was not associated with HT in the Japanese population, suggesting that other SNP(s) of the Tg gene may be associated with AITD [24]. Therefore, we now tested all reported Tg SNPs in our cohort of Japanese AITD patients.

Tg is one of the three genes encoding major disease-specific thyroid autoantigens, including also the thyroid peroxidase (TPO) and TSH receptor (TSHR) genes. The association of Tg variants with AITD demonstrates that thyroid specific genes, and not only immune regulatory genes (e.g. HLA-DR, CTLA-4), are important for susceptibility to AITD. Moreover, our results suggest that the association of Tg with AITD is not specific to one population and is observed across ethnic backgrounds, as had been shown for CTLA-4 [29] and CD40 [30].

Polymorphisms in Tg gene have previously been studied in different ethnic groups, US Caucasians [16], [23], UK Caucasians [22], [31] and Japanese [24], making it possible to compare the association of some of the polymorphisms in different ethnic groups. For example, a microsatellite polymorphism in intron 27 (Tgms2) was studied in all three ethnic groups and significantly association with AITD was reported in all studies [16], [22], [24]. In contrast, exon 10–12 and exon 33 SNPs were significantly associated with AITD in US dataset [23], but not in UK [31] or Japanese (present study) datasets. Therefore, it is possible that the genetic susceptibility to AITD involves both different polymorphisms in the same gene in different ethnic/geographic groups, as well as common polymorphism that predisposes to AITD in different ethnic/geographic groups. Tgms2 may be the common polymorphism across ethnic and geographic groups.

It is likely that susceptibility to AITD involves an interaction between several genes, including immune regulatory genes and tissue specific genes, as well as environmental factors. Indeed, previous analysis showed evidence for interaction between HLA-DR3 and a Tg exon 33 SNP [23]. Recently, Hodge et al. [32] demonstrated a possible interaction between the effects of inheriting at least one copy of the DRβ-Arg74 allele (R) of the DRB1 gene and inheriting the homozygous CC genotype of the exon 33 SNP. This proposed mechanism of immune-regulatory genes interacting with autoantigen specific genes, may be a more general mechanism for the development of organ-specific autoimmune diseases. This mechanism has been shown to play a role in the etiology of Type 1 (autoimmune) diabetes (T1D) [33]. Possible mechanism for the biological basis of these interactions is that the susceptibility SNPs in Tg predispose to AITD by influencing the formation of immunogenic peptides and their presentation by HLA-DR3 to T-cells. However, further structural-functional studies are required to substantiate this model [34].

The same 12 SNPs used in the present study were previously studied in US Caucasian population, and exon 10–12 SNP cluster and an exon 33 SNP were reported to be significantly associated with both GD and HT, with a haplotype consisting of these two SNP groups more strongly associated with and a gene-gene interaction between HLA-DR3 and the exon 33 SNP suggested [23]. However, these SNPs were not associated with the disease and instead different SNPs in intron 41 were associated with GD, but not HT, in the present study. Given the difference in HLA between Japanese and Caucasian populations, different genetic interaction between Tg peptides and HLA class II pockets may be responsible for the differing Tg SNPs that are associated with AITD in Japanese and Caucasian populations.

In conclusion, our results suggest that Tg is a susceptibility gene for AITD and GD in the Japanese population. Therefore, it is possible that that the Tg gene may predispose to AITD across populations of different ethnic backgrounds. However, at this stage, we cannot exclude that the 8q24 region harbors another susceptibility locus (i.e. SAS-ZFAT) for AITD in linkage disequilibrium with those SNPs of the Tg gene.

## Supporting Information

Table S1 The pair-wise LD between 25 SNPs in the Tg gene
(PDF)
