# Supplementary material for: Multiple SNPs in Intron 41 of Thyroglobulin Gene Are Associated with Autoimmune Thyroid Disease in the Japanese Population
Source: PLoS One. 2012 May 25;7(5):e37501. doi: 10.1371/journal.pone.0037501 (PMC3360768; doi:10.1371/journal.pone.0037501)
Supplement: Table S1 — The pair-wise LD between 25 SNPs in the Tg gene (PDF) [file pone.0037501.s001.pdf]

Table S1. The pair-wise LD between 25 SNPs in the Tg gene

SNP No.	SNP Name	Allele	Chromosome position in NT_008046.15	Location	LD (r^2)																							
					[01]	[02]	[03]	[04]	[05]	[06]	[07]	[08]	[09]	[10]	[11]	[12]	[13]	[14]	[15]	[16]	[17]	[18]	[19]	[20]	[21]	[22]	[23]	[24]
[01]	-1984[a]	C/T	unknown	Promoter																								
[02]	-1714[a]	G/A	unknown	Promoter	0.299																							
[03]	rs180223 (E10SNP24[b])	G/T	133969434	Exon 10	0.102	0.256																						
[04]	rs2069550 (E10SNP158 [b])	C/T	133969568	Exon 10	0.086	0.275	0.944																					
[05]	rs853326 (E12SNP [b])	G/A	133979156	Exon 12	0.104	0.251	0.915	0.919																				
[06]	rs2068128	C/T	133986770	Intron 16	0.278	0.100	0.010	0.006	0.011																			
[07]	rs2261147 (E18SNP-20 [b])	T/C	133989593	Intron 17	0.323	0.097	0.026	0.020	0.031	0.577																		
[08]	rs2069556 (E18SNP88 [b])	A/G	133989700	Exon 18	0.322	0.109	0.020	0.015	0.025	0.554	0.921																	
[09]	rs853304 (E21SNP [b])	T/C	134000930	Exon 21	0.336	0.109	0.025	0.019	0.030	0.600	0.962	0.938																
[10]	rs2246624 (E27SNP [b])	T/C	134030173	Intron 26	0.033	0.063	0.236	0.232	0.240	0.043	0.098	0.090	0.102															
[11]	rs2069561 (E29SNP [b])	G/A	134044465	Exon 29	0.326	0.117	0.015	0.010	0.021	0.499	0.835	0.812	0.858	0.109														
[12]	rs7829428	A/G	134048293	Intron 30	0.107	0.205	0.049	0.054	0.042	0.342	0.321	0.316	0.345	0.120	0.367													
[13]	rs2687809	G/A	134050687	Intron 31	0.015	0.003	0.003	0.003	0.003	0.001	0.003	0.001	0.002	0.004	0.001	0.001												
[14]	rs2076740 (E33SNP [b])	C/T	134053240	Exon 33	0.022	0.002	0.005	0.007	0.005	0.003	0.000	0.000	0.000	0.009	0.000	0.005	0.028											
[15]	rs10505604	A/G	134096770	Intron 37	0.019	0.006	0.000	0.000	0.000	0.000	0.002	0.002	0.003	0.001	0.003	0.002	0.022	0.374										
[16]	rs3739266	T/C	134119860	Intron 41	0.011	0.008	0.001	0.001	0.000	0.000	0.001	0.001	0.002	0.000	0.003	0.001	0.015	0.267	0.793									
[17]	rs2256366	T/C	134126284	Intron 41	0.000	0.007	0.007	0.007	0.005	0.000	0.000	0.000	0.000	0.000	0.001	0.000	0.029	0.037	0.084	0.112								
[18]	rs2253035	G/C	134130242	Intron 41	0.008	0.014	0.002	0.001	0.001	0.000	0.000	0.001	0.000	0.004	0.001	0.000	0.005	0.093	0.297	0.353	0.269							
[19]	rs2687836	C/T	134131448	Intron 41	0.000	0.007	0.008	0.008	0.008	0.000	0.000	0.001	0.000	0.000	0.002	0.001	0.022	0.030	0.070	0.095	0.793	0.315						
[20]	rs2252696	C/A	134132714	Intron 41	0.004	0.000	0.005	0.005	0.004	0.004	0.000	0.000	0.000	0.001	0.002	0.002	0.007	0.000	0.007	0.004	0.204	0.023	0.091					
[21]	rs1124527	A/C	134143287	Intron 41	0.002	0.005	0.003	0.003	0.003	0.000	0.000	0.000	0.000	0.000	0.001	0.002	0.027	0.003	0.016	0.025	0.450	0.094	0.340	0.310				
[22]	rs2256476	T/G	134176318	Intron 41	0.005	0.001	0.000	0.000	0.000	0.002	0.006	0.009	0.007	0.001	0.003	0.001	0.021	0.008	0.008	0.013	0.425	0.046	0.316	0.184	0.618			
[23]	rs2069568 (E43SNP4 [b])	C/T	134177635	Exon 43	0.004	0.001	0.000	0.000	0.000	0.002	0.003	0.004	0.003	0.000	0.001	0.000	0.018	0.003	0.002	0.005	0.279	0.008	0.184	0.164	0.419	0.707		
[24]	rs2069569 (E43SNP97 [b])	T/C	134177728	Exon 43	0.002	0.001	0.001	0.001	0.002	0.008	0.010	0.010	0.009	0.002	0.003	0.001	0.014	0.000	0.018	0.013	0.194	0.054	0.086	0.302	0.203	0.401	0.550	
[25]	rs2294024 (E46SNP [b])	C/T	134213295	Exon 46	0.000	0.002	0.001	0.002	0.001	0.003	0.001	0.001	0.001	0.004	0.001	0.001	0.005	0.007	0.056	0.067	0.052	0.193	0.070	0.010	0.130	0.162	0.239	0.034

[a]SNP numbers corresponding to those in reported by Stefan et al. (34).

[b]SNP numbers corresponding to those in reported by Ban et al. (23).
